# Effects of Air Temperature on Transient Expression of Influenza Hemagglutinin in *Nicotiana benthamiana*: Analysis of Transgene Transcription and Plant Stress Responses

**DOI:** 10.1002/bit.28942

**Published:** 2025-01-31

**Authors:** Patthasarun Pruksarojanakul, Kazune Atsumi, Youngjun Oh, Nobuyuki Matoba, Ryo Matsuda

**Affiliations:** ^1^ Department of Biological and Environmental Engineering, Graduate School of Agricultural and Life Sciences The University of Tokyo Tokyo Japan; ^2^ Department of Pharmacology and Toxicology University of Louisville School of Medicine Louisville Kentucky USA

**Keywords:** endoplasmic reticulum stress, hypersensitive response, plant‐made pharmaceuticals, recombinant influenza hemagglutinin, temperature effects, transient expression

## Abstract

Postinfiltration air temperature is known to affect the accumulation of recombinant protein in *Agrobacterium*‐mediated transient expression in *Nicotiana benthamiana* plants, including the number of days needed to reach maximum content and the rate of reduction thereafter. This study aimed to clarify whether the transcript levels of the transgenes and those of plant stress response markers (i.e., hypersensitive response and endoplasmic reticulum [ER] stress) could be primary determinants of the accumulation of recombinant influenza hemagglutinin (HA) at 21 or 26°C. We found no correlation between the transgene expression levels (*HA, RdRP*, and *MP*) and the number of days needed to reach the maximum HA protein content at both temperatures. Regardless of the accumulation compartment, HA protein content peaked earlier at 26°C than at 21°C. The rapid reduction of HA content after reaching the maximum, observed only in the ER at 26°C, correlated with severe necrosis and high transcript levels of two representative ER stress markers, *bZIP60* and *BiP*. This correlation suggests that high postinfiltration air temperature affects HA accumulation primarily through ER stress, a key factor in the rapid reduction of HA content after the peak.

## Graphical abstract

Effects on the number of days required to reach the maximum hemagglutinin (HA) content did not solely depend on the transgene transcript levels, regardless of the accumulation compartment. Effects on the reduction rate after the maximum HA content correlated with endoplasmic reticulum stress response, depending on the accumulation compartment.

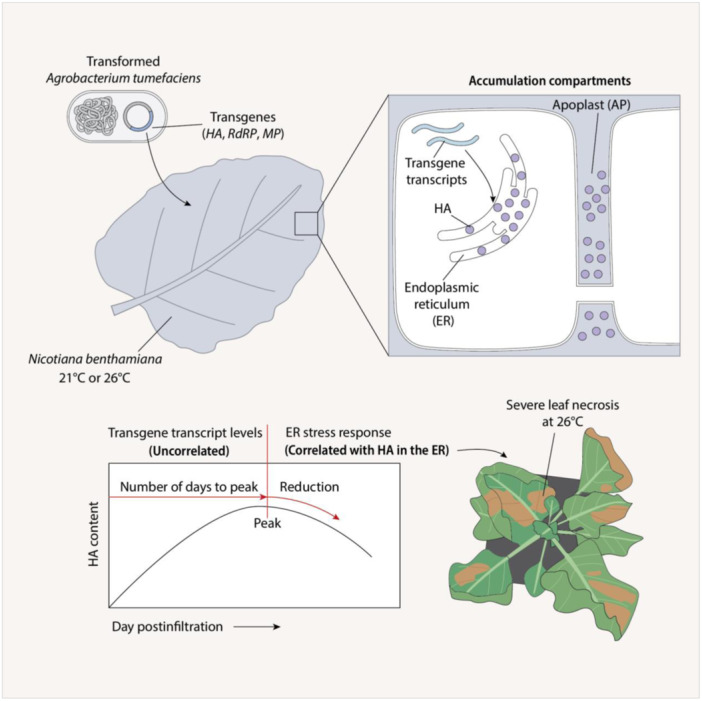

## 1 Introduction

Transient expression (TE) in whole plants is a method that allows a rapid production of biopharmaceutical proteins for medical uses in large scale (Eidenberger, Kogelmann, and Steinkellner 2023; Fausther‐Bovendo and Kobinger 2021; Lee et al. 2024; Margolin et al. 2020; Marillonnet et al. 2005; Oh et al. 2023; Siriwattananon et al. 2021; Ward et al. 2021). Compared to mammalian and bacterial cell cultures, whole plants offers several advantages to recombinant protein production such as higher upstream production scalability, lower upstream production cost, and lower risk of contamination with human or animal pathogens (Chincinska 2021; Eidenberger, Kogelmann, and Steinkellner 2023; Fausther‐Bovendo and Kobinger 2021; Gleba, Klimyuk, and Marillonnet 2007; Klimyuk et al. 2014; Margolin et al. 2020; Marillonnet et al. 2005; Matoba, Davis, and Palmer 2011; Siriwattananon et al. 2021; Ward et al. 2021). Among the TE methods using whole plants, the one employing *Agrobacterium tumefaciens* (hereafter referred to as the *Agrobacterium*‐mediated TE) to introduce transgenes into the tobacco plants (*Nicotiana tabacum*) or its relative (*N. benthamiana*) (Chincinska 2021; Gleba, Klimyuk, and Marillonnet 2007; Klimyuk et al. 2014; Matoba, Davis, and Palmer 2011; Marillonnet et al. 2005) is currently employed by several manufacturers (e.g., PlantForm [Canada]; Aramis Biotechnologies [Canada]; Baiya Phytopharm [Thailand]; KBio (USA); BioApplications [Korea]; Agrenvec [Spain]; Icon Genetics GmbH [Germany]).

Air temperature is one of the environmental factors involved in plant cultivation that significantly affects the recombinant protein content in the TE (Buyel and Fischer 2012; Cazzonelli and Velten 2006; De Clercq et al. 2002; Dillen et al. 1997; Fujiuchi et al. 2016; Kondo, Hasegawa, and Suzuki 2000; Joh et al. 2005; Jung, McDonald, and Dandekar 2015; Matsuda, Abe, and Fujiwara 2017a; 2017b; 2018; Moon et al. 2014). Previous research demonstrated that air temperature after introducing transgenes into the plants (hereafter referred to as postinfiltration temperature) could impact two characteristics of the accumulation of recombinant influenza hemagglutinin (HA) in the endoplasmic reticulum (ER) and plant health (Matsuda et al. 2017a). These characteristics are the number of days required to reach the peak HA content and the reduction rate of HA content after the peak (Matsuda et al. 2017a). Compared to 21°C, 25°C reduced the number of days required to reach the maximum HA content. However, 25°C led to a rapid reduction of HA content after the peak, along with severe leaf necrosis (Matsuda et al. 2017a). Ideally, the reduction rate after the peak should be slow to reduce the chance of missing high recombinant protein yield at harvest, and the extent of necrosis should be minimized to reduce the cost of removing secondary metabolites accumulated in necrotic leaves (Al‐Khayri et al. 2023; Robert et al. 2015; Wilken and Nikolov 2012). That research highlights a problem that targeting HA to the ER, which is a strategy commonly used to achieve high recombinant protein content (Benchabane et al. 2008; Feng et al. 2022; Song et al. 2024), could increase HA content compared to targeting to the apoplast (AP). However, targeting HA to the ER could result in the undesirable effects when plants were cultivated at 25°C to achieve the peak quickly (Matsuda et al. 2017a). Understanding the reasons behind the postinfiltration effects on the course of recombinant protein accumulation and plant health would pave the way to developing a production method to achieve high recombinant protein content quickly with slow reduction after the peak.

Thus, this research aimed to clarify a part of the reasons behind the postinfiltration effects on the course of recombinant protein accumulation (i.e., the number of days needed to reach the maximum HA content, the HA content at the maximum, and the reduction rate after the maximum HA) and plant health. Fundamentally, the recombinant protein content depends on its synthesis and degradation (Egelkrout, Rajan, and Howard 2012). Therefore, we set two hypotheses that the postinfiltration temperature might influence the HA accumulation through the synthesis and degradation of HA as follows.

On the synthesis side, we hypothesized that the number of days needed to reach the maximum HA content and/or the HA content at the peak might be correlated with the effects of postinfiltration temperature on the maximum transcript levels of the transgenes and/or the number of days required to reach the maximum levels. It is generally known that high gene expression does not always result in high protein yields, as various factors affect protein levels posttranscriptionally (Liu and Nelson 2013). However, it remains unclear whether this principle also applies to *Agrobacterium*‐mediated TE of recombinant proteins in *N. benthamiana*. Since the current research hypothesis was derived from the previous research (Matsuda et al. 2017a), we continued using the same protein model (HA). We investigated the transcript levels of three transgenes in a tobamovirus (TMV)‐based deconstructed viral system (magnICON®) (Klimyuk et al. 2014; Marillonnet et al. 2004), including the gene encoding HA protein (*HA*), the RNA‐dependent RNA polymerase gene (*RdRP*), and the movement protein gene (*MP*) (Matsuda et al. 2012; 2017a; Marillonnet et al. 2004). The latter two support the synthesis of HA protein, that is, *RdRP* amplifies the transcripts of all transgenes, while *MP* promotes the movement of the transgene transcripts to neighboring cells (Liu and Nelson 2013; Lucas, Ham, and Kim 2009).

On the degradation side, we set the hypothesis based on the previous work reporting that necrosis correlated with low recombinant protein yields (Hamorsky et al. 2015; Nosaki et al. 2021), and severe necrosis was observed along with a rapid reduction rate of HA content (Matsuda et al. 2017a). Here, we clarified the effects of accumulation compartments on the impact of postinfiltration temperature on the transcript levels of transgenes and marker genes involved in plant response to ER stress and hypersensitive response (HR). We hypothesized that the reduction rate after the maximum HA content might be correlated with the severity of leaf necrosis. Leaf necrosis observed during the accumulation of HA might be caused by plant responses associated with the ER stress (Hamel et al. 2024; Hamorsky et al. 2015; Nosaki et al. 2021) and/or the HR (Hamorsky et al. 2015). We observed the ER stress using transcript levels of marker genes, including *bZIP60* (Cao et al. 2022; Hamorsky et al. 2015; Zhang and Wang 2016), *BiP* (Hamorsky et al. 2015), and *PDI* (Hamorsky et al. 2015; Zhang and Wang 2016). We monitored the HR using transcript levels of its marker gene, *PR1a* (Hamorsky et al. 2015).

In this research, we also aimed to clarify the impact of the temperature effects on the correlation between the transcript levels and the HA content depended on accumulation compartments (the ER or the AP). We analyzed the impact of two postinfiltration temperature levels (21°C and 26°C) on HA accumulation by comparing HA content at these temperatures across the same phases (i.e., before reaching the peak, at the peak, and after the peak). Because of the difference in thermal radiation of different light sources (Matsuda, Abe, and Fujiwara 2017b), we set all air temperature levels to 1°C higher (21°C and 26°C) than those in a previous study (20°C and 25°C) (Matsuda et al. 2017a) to make leaf temperature levels comparable to those of the previous research (Matsuda et al. 2017a). Our previous work showed that HA content peaked earlier at 25°C than at 21°C (Matsuda et al. 2017a); we, therefore, specified a sampling window for each postinfiltration temperature level to investigate HA content across the three accumulation phases. Alongside HA content, we quantified total soluble protein (TSP) content at 21°C and 26°C from 5 to 8 dpi and 3 to 6 dpi, respectively. The TSP is the amount of protein in a leaf sample that dissolves in the liquid phase. In plant‐made pharmaceutical protein production, the TSP is often used as an indicator of the overall protein level in host plants (e.g., Fujiuchi et al. 2016, Matsuda et al. 2017a). Measuring TSP content provides valuable information for those engaged in plant‐made pharmaceutical research and helps us understand the dynamics of overall protein in response to temperature changes, complementing our analysis of specific changes in HA protein content.

## 2 Results

### 2.1 Effects of Postinfiltration Temperature on the HA and TSP Contents

**Figure 1:**
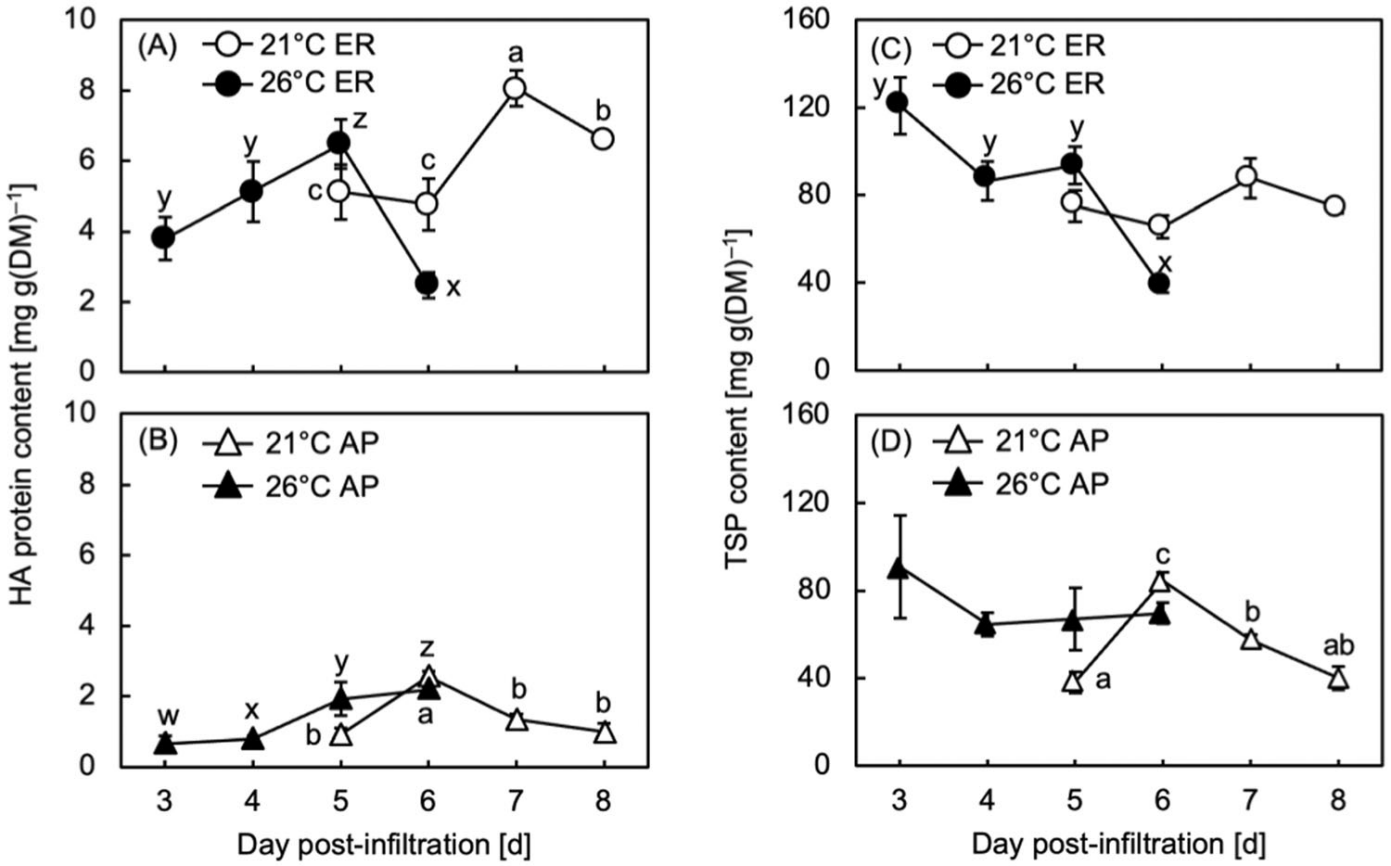
(A–B) Time courses of hemagglutinin (HA) protein content per unit dry mass (DM) in (A) the endoplasmic reticulum (ER) and (B) the apoplast (AP) in leaves of *Nicotiana benthamiana* plants grown at a postinfiltration air temperature of 21°C (open symbols) or 26°C (closed symbols). (C–D) Time courses of total soluble protein (TSP) content per unit dry mass (DM) in (C) the endoplasmic reticulum (ER) and (D) the apoplast (AP) in leaves of *Nicotiana benthamiana* plants grown at a postinfiltration air temperature of 21°C (open symbols) or 26°C (closed symbols). The symbols represent the means of biological triplicates (*n* = 3). The vertical bars represent the standard errors of the means. In each panel, different small letters indicate a significant difference in means among days post‐infiltration (dpi) within the same treatment (Tukey's HSD test at *p* < 0.05).

Figure 1A,B show that the HA content in the ER peaked 2 days earlier at 26°C (5 dpi) than at 21°C (7 dpi) (Figure 1A). After reaching the peak, it decreased sharply at 26°C but decreased relatively slowly at 21°C. When HA was localized in the AP, the effects on both the number of days required to reach the peak and the reduction rate of the HA content were again observed. The maximum HA content in the AP at 21°C and 26°C (Figure 1B) was approximately 25%–30% of the levels in the ER (Figure 1A). A rapid increase in HA content in the AP was detected 1 day later at 21°C (from 5 to 6 dpi) than at 26°C (from 4 to 5 dpi) (Figure 1B). The HA content in the AP peaked at 6 dpi at both temperature levels (Figure 1B).

Figure 1C,D showed data on TSP contents. They showed that the changes in the TSP content did not show a clear correlation with the HA content. The changes in TSP content did not correspond to those of the HA content in the ER over time at both 21°C and 26°C (Figure 1A). When HA was localized in the AP at 26°C, no significant change in the TSP content was observed over the sampling window from 3 to 6 dpi (Figure 1D), while a gradual increase in HA content in the AP was observed over the same period. In contrast, both the TSP (Figure 1D) and HA (Figure 1B) contents in the AP showed similar trends at 21°C, reaching their maximum at 6 dpi.

### 2.2 Leaf Necrosis

**Figure 2:**
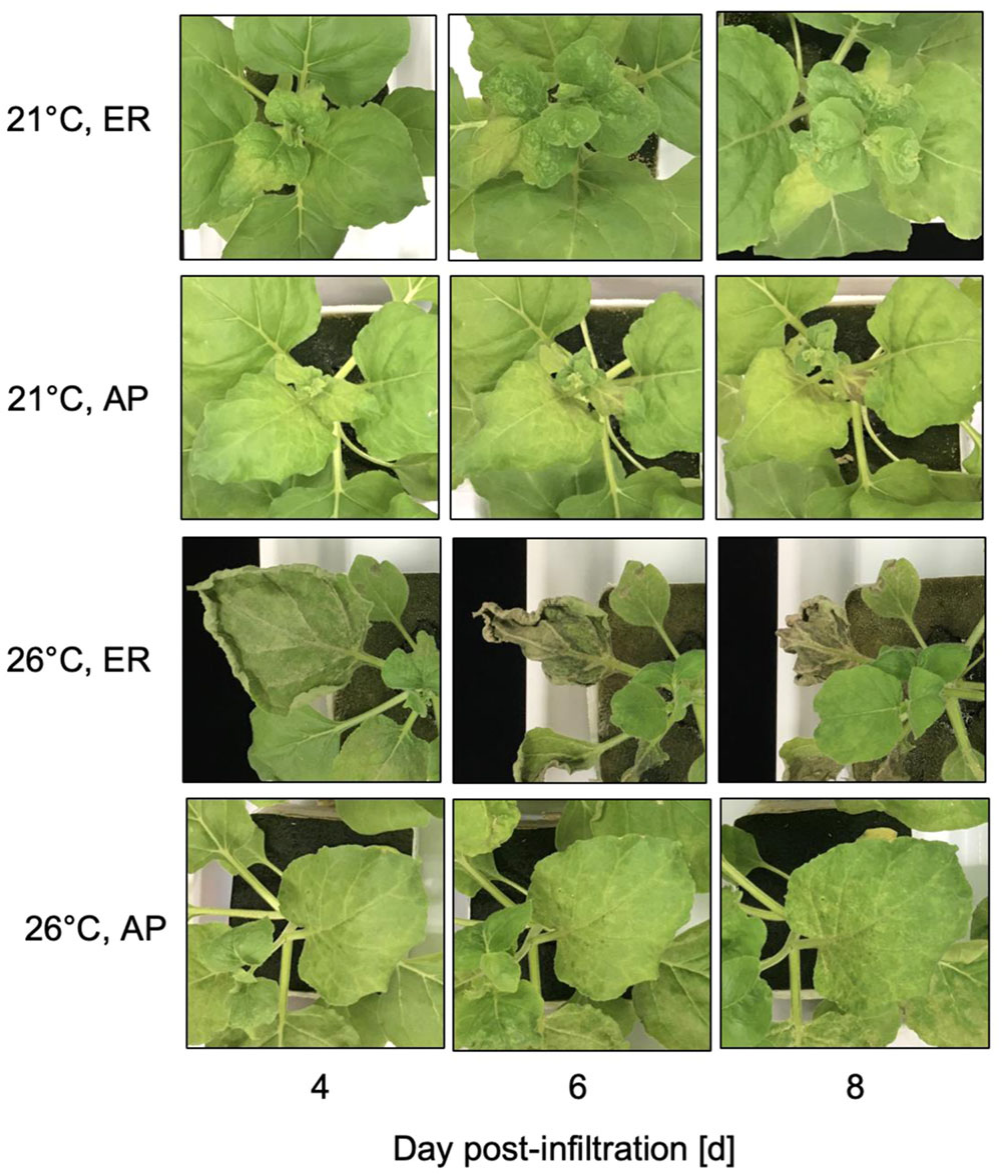
Appearance of *Nicotiana benthamiana* leaves that accumulated hemagglutinin (HA) in the endoplasmic reticulum (ER) and in the apoplast (AP) grown at a postinfiltration air temperature of 21°C or 26°C at 4, 6 and 8 days postinfiltration (dpi).

**Figure 3:**
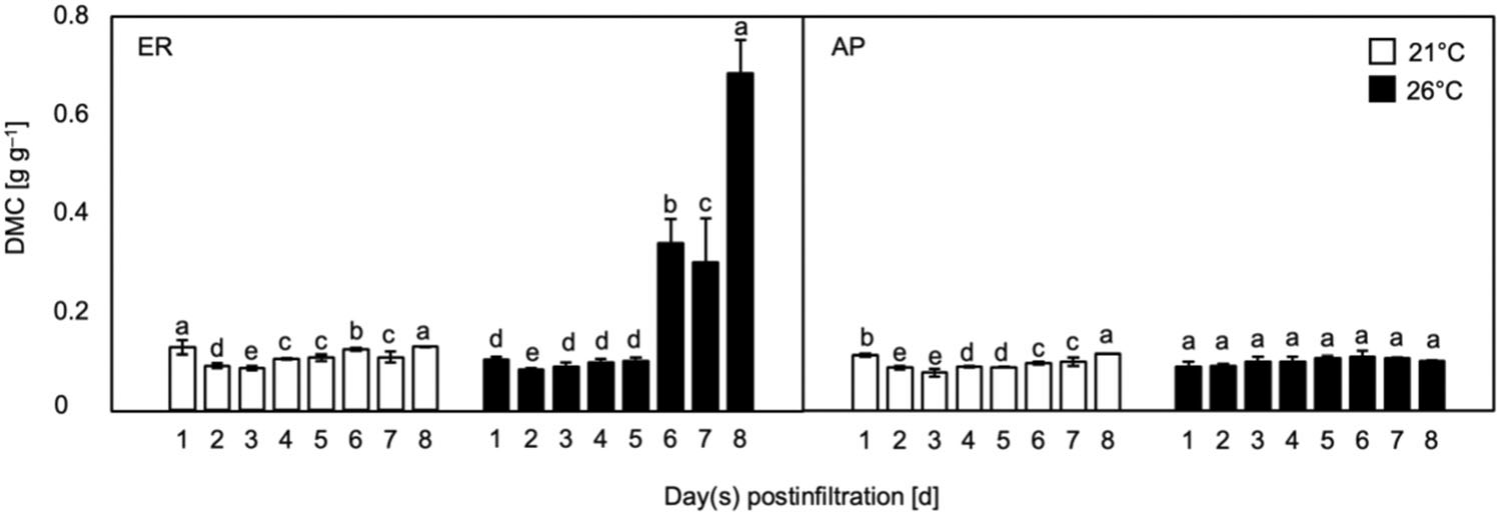
Time courses of the dry matter content (DMC) of *Nicotiana benthamiana* leaves accumulating hemagglutinin (HA) protein in the endoplasmic reticulum (ER) or the apoplast (AP) and grown at a postinfiltration air temperature of 21°C or 26°C. Data represent the means of biological triplicates (*n* = 3). The vertical bars represent the standard errors of the means. Different small letters indicate a significant difference in means among days post‐infiltration (dpi) within the same treatment (Tukey's HSD test at *p* < 0.05).

Leaves localizing HA in the ER at 26°C exhibited necrosis to the greatest extent among all treatments. Severe leaf dehydration and dead leaf tissue were observed in almost the entire leaf lamina from 4 dpi (Figure 2). The time courses of leaf dry matter content (DMC), a proxy for quantitatively evaluating the degree of necrosis used in our previous research (Matsuda et al. 2017a), were investigated. The DMC was almost stable from 1 to 8 dpi when HA was accumulated in the ER at 21°C and in the AP at 21°C and 26°C (Figure 3). Only when HA was accumulated in the ER at 26°C, a significant increase in the DMC was detected after 5 dpi (Figure 3).

### 2.3 Transcript Levels of the Transgenes (*HA, RdRP*, and *MP*)

**Figure 4:**
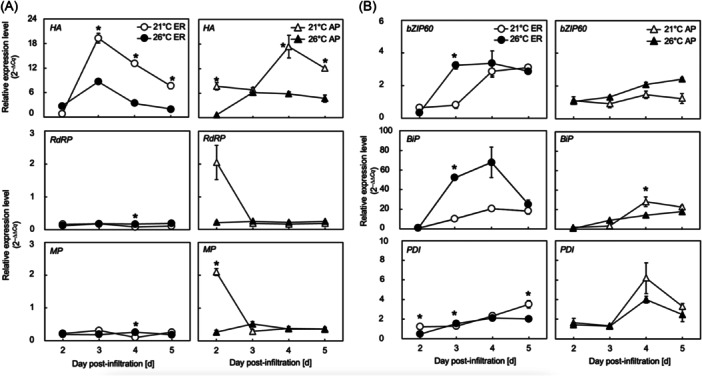
Time courses of transcript levels of representative marker genes in leaves accumulating HA in the endoplasmic reticulum (ER) or the apoplast (AP) of *Nicotiana benthamiana* plants grown at 21°C (open symbols) or 26°C (closed symbols) at 2–5 days post‐infiltration (dpi). (A) The expression involved in the synthesis of hemagglutinin (HA) protein, including *HA*, *RdRP*, and *MP*, and (B) those of the endoplasmic reticulum (ER) stress, including *bZIP60, BiP*, and *PDI*. The symbols represent the means of biological triplicates (*n* = 3). The vertical bars represent the standard errors of the means. In each panel, the asterisks indicate a significant difference in means at the same dpi between 21°C and 26°C (Welch's *t*‐test, *p* < 0.05).

We measured the transcript levels of the transgenes, including *HA*, *RdRP*, and *MP*, by RT‐qPCR analysis using total RNA extracted from leaf tissues. When HA was localized in the ER, the transcript level of *HA* peaked at 3 dpi at both temperature levels (Figure 4A). The maximum transcript level of *HA* was approximately three times greater at 21°C than at 26°C (Figure 4A). The maximum transcript levels of *RdRP* and *MP* were almost comparable between 21°C and 26°C (Figure 4A).

When HA was localized in the AP, the maximum transcript levels of *HA* were comparable to those observed in leaves localizing HA in the ER at both temperature levels (Figure 4A). However, the maximum transcript level of *HA* for localizing HA in the AP (Figure 4A) was reached 1 d later than that for localizing HA in the ER at 21°C (Figure 4A). The transcript levels of *RdRP* and *MP* in leaves localizing HA in the AP were almost comparable between 21°C and 26°C at 3, 4, and 5 dpi (Figure 4A), except at 2 dpi when the transcript levels was greater at 21°C than at 26°C (Figure 4A).

### 2.4 Transcript Levels of ER Stress (*bZIP60, BiP*, and *PDI*) and HR (*PR1a*) Markers

When HA was localized in the ER, the expression of *bZIP60* and *BiP* markedly increased at 26°C after 2 dpi, and the high transcript levels of these genes continued until 4 or 5 dpi (Figure 4B). Unlike those of *bZIP60* and *BiP*, the RNA expression levels of *PDI* increased gradually over time from 2 to 5 dpi at both 21°C and 26°C (Figure 4B). Although the transcript levels of *PDI* were significantly different between the two temperature groups at some dpi, they were almost comparable at 3 and 4 dpi at both temperature levels (Figure 4B).

When HA protein was localized in the AP, the transcript levels of *bZIP60* and *BiP* gradually increased over at both 21°C and 26°C with no significant difference between the two temperature levels at almost all dpi (Figure 4B). At 26°C, no peaks in the transcript levels of *bZIP60* and *BiP* were detected during the period of 2–5 dpi, while at 21°C, transcript levels of these genes peaked at 4 dpi (Figure 4B). The expression of *PDI* peaked at 4 dpi at both 21°C and 26°C, but the transcript levels were not significantly different between different temperatures on the same day.

**Figure 5:**
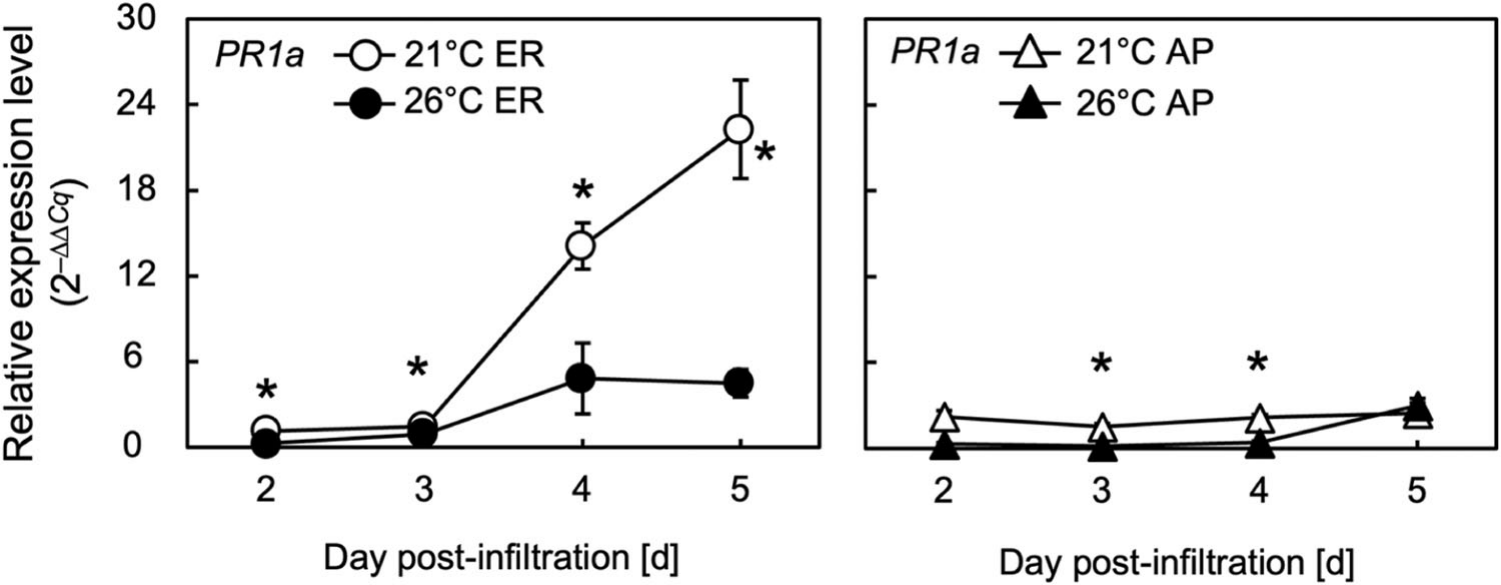
Time courses of transcript levels of a representative marker gene involved in the hypersensitive response (HR), *PR1a*, in leaves accumulating HA in the endoplasmic reticulum (ER) or the apoplast (AP) of *Nicotiana benthamiana* plants grown at 21°C (open symbols) or 26°C (closed symbols) at 2–5 days post‐infiltration (dpi). The symbols represent the means of biological triplicates (*n* = 3). The vertical bars represent the standard errors of the means. In each panel, the asterisks indicate a significant difference in means at the same dpi between 21°C and 26°C (Welch's *t*‐test, *p* < 0.05).

The transcript levels of the HR marker *PR1a* (Figure 5) in leaves in which HA protein was localized in the ER started to increase rapidly after 3 dpi, with overall expression levels lower at 26°C than at 21°C from 2 to 5 dpi (Figure 5). In leaves localizing HA in the AP, the expression levels of *PR1a* remained relatively low throughout the sampling window and were slightly greater at 21°C than at 26°C (Figure 5).

## 3 Discussion

This research confirmed the effects of postinfiltration temperature, that is, 21°C and 26°C, on the number of days needed for HA protein content to peak and its subsequent reduction rate in the ER, which were similar to those observed previously using the same expression system and plant model at 20°C and 25°C (Matsuda et al. 2017a). As for the number of days needed for HA protein content to peak, we further clarified that the effects of temperature on the number of days for HA protein content to peak varied slightly depending on the accumulation compartment in the leaf tissues. However, the overall trend was that HA protein content peaked earlier at 26°C than at 21°C, regardless of the accumulation compartments. From a practical point of view, there is still a need to balance trade‐offs in production and to evaluate plant response to temperature settings on a case‐by‐case basis. This research highlights a trade‐off in using temperature control to speed up high yield versus the risk of invoking severe necrosis (Matsuda et al. 2017a). Necrosis is undesirable as it can cause the accumulation of secondary metabolites in leaves, which complicates downstream purification processes. It is important to note that constant postinfiltration temperature control applied throughout the day and night in this experiment may differ from conditions in production facilities where day‐night temperature variations are implemented. Although the overall trends regarding the HA accumulation characteristics, transcript levels, and ER stress response could be extrapolated to be similar to our findings if the day average temperature is consistent, the actual response of plants should be verified with experimental data.

The impact of temperature on the changes in HA and TSP contents differed depending on the temperature levels and accumulation compartment in leaf tissues. At 26°C, the trends of TSP content changing over time did not match with the changes in HA content before reaching the peak in both accumulation compartments. After the peak HA content, the trends of TSP matched with HA contents, regardless of temperature levels and accumulation compartments. This suggests that temperature affected the reduction of both the overall soluble protein and HA protein, regardless of accumulation compartments. Since the protein content depends on the balance between its synthesis and degradation rates, we investigated whether transgenes for HA protein synthesis and representative marker genes for HR and ER stress could be the main determinants.

For the synthesis side of our hypothesis, we investigated how temperature affected the transcript levels of transgenes (*HA*, *RdRP*, and *MP*) and correlated them with the time course of HA content at the respective temperature levels. Temperature did not affect the HA protein content solely through the transcript levels of the transgenes involved in the synthesis of HA protein, all of which were the transgenes introduced into the plant at the time of agroinfiltration. This result was confirmed by the following evidence. First, a clear association between their maximum transcript levels and the maximum HA protein content in the ER was not detected. The difference in the maximum HA content between the two temperature groups was only 30% (approximately 8 and 6 mg g(DM)^–1^ at 21°C and 26°C, respectively), while the relative *HA* expression levels were approximately two times greater at 21°C than at 26°C. Second, the number of days needed to reach the maximum transcript levels of these genes did not correspond with the number of days needed to reach the maximum HA protein content. The transcript levels of *HA* peaked on the same day at both temperature levels, while the HA protein content peaked 2 d earlier at 26°C than at 21°C.

It was noted that *RdRP* and *MP* were overexpressed at 2 dpi when HA was localized in the AP at 21°C. The exact cause of this overexpression is currently unknown. Despite the overexpression of *RdRP* and *MP*, our conclusion remains valid that transgene transcript levels were not the primary determinant of the postinfiltration temperature effects on the number of days to the maximum HA content and the peak levels of HA content.

For the degradation side of our hypothesis, we found that some of the representative genes involved in ER stress showed a significant association with the high reduction rate of HA content in the ER at 26°C. The transcript levels of *bZIP60* and *BiP* at 26°C, which were higher than those at 21°C within a few days (after 2 dpi) after agroinfiltration, were approximately the same as the levels at which the transcript levels of *HA* significantly increased, suggesting that the expression of *HA* led to a stronger unfolded protein response (UPR) at 26°C than at 21°C. Moreover, the high transcript levels of these two marker genes were followed by a significant increase in leaf DMC (after 5 dpi) at 26°C. This result suggests that severe necrosis is partly due to high ER stress levels. We found that the progression of leaf necrosis came before the rapid change in DMC could be detected. This suggests that leaf dehydration and dryness were negligible in early necrosis. DMC is a good qualitative indicator to compare the extent of necrosis about 2 days after necrosis can be visualized.

The ER stress commonly occurs during the expression of biopharmaceutical proteins and is known to be induced by the accumulation of unfolded and misfolded proteins in the ER. Using the same expression vector and host plant used in the present research to produce the chlorella toxin B subunit (CTB), researchers previously demonstrated that increased transcript levels of *bZIP60*, *BiP*, and *PDI* occurred along with the degradation of misfolded and unfolded CTB polypeptides through the ER‐associated degradation pathway (Hamorsky et al. 2015). Moreover, it was confirmed that the expression of recombinant influenza hemagglutinin H5 in *N. benthamiana* resulted in the accumulation of UPR proteins due to ER stress (Hamel et al. 2024). The expression of the BiP protein increased as necrosis occurred in *N. benthamiana* leaves expressing human F‐box and human Cul1 proteins (Nosaki et al. 2021). Therefore, the high transcript levels of *bZIP60* and *BiP* observed in the present study suggest that the rapid decrease in the HA protein content in the ER at 26°C is due to the degradation of HA polypeptides in response to high ER stress. Unlike that of *bZIP60* and *BiP*, the expression of *PDI* increased gradually at 21°C and 26°C, indicating that *PDI* transcript levels did not change in relation to the reduction in HA protein content at either temperature. Thus, its transcript levels was not the main determinant of the reduction rate after the HA protein content reached its peak.

The transcript level time courses of the representative HR marker gene, *PR1a*, in the leaves of HA protein in the ER showed that *PR1a* expression was influenced by temperature, and the overall expression was greater at 21°C than at 26°C. The underlying mechanism behind the upregulation of the *PR1a* expression when HA was localized in the ER at 21°C remains unknown. Considering the transcript level time course along with the severity of necrosis, severe necrosis in plants with HA localized in the ER at 26°C did not correlate with HR.

For the degradation side of our hypothesis, we confirmed that the effects of temperature on the representative genes involved in the temperature effect on the reduction rate of HA content were specific to the HA protein accumulation compartment in the leaf tissue. Changing the accumulation compartment from the ER to the AP reduced the extent of ER stress in leaves with accumulated HA protein, particularly at 26°C. This may explain why severe leaf necrosis was not observed in leaves with accumulated HA protein in the AP treatment at 26°C. Although the extent of ER stress was relatively low in leaves that accumulated HA protein in the AP compared to that in the ER, the HA protein content in the AP was low. The low HA protein content in the AP may result from several factors, such as leaf proteases, during the posttranslation of HA (Goulet et al. 2012; Grosse‐Holz et al. 2018; Jutras, Dodds, and van der Hoorn 2020).

In conclusion, this research highlights the significant impact of postinfiltration air temperature on the accumulation of the recombinant protein during *Agrobacterium*‐mediated transient expression. We confirmed that postinfiltration temperature did not affect the number of days needed to reach the maximum HA content through the expression levels of the transgenes. Then, we confirmed that the reduction rate after the maximum was correlated with high transcript levels of the representative ER stress markers (i.e., *bZIP60* and *BiP*) when HA was accumulated in the ER at 26°C. Therefore, temperature after agroinfiltration does not affect recombinant protein accumulation through the expression levels of the transgenes but likely through posttranscription events such as ER stress response.

It is possible that variation in recombinant protein structures could affect the extent of plant stress response during the transient expression (Hamorsky et al. 2015). Future investigation using various protein models may offer deeper insights into the relationship between postinfiltration temperature and recombinant protein expression under various biotic stress levels. The expression time courses of the transgenes and stress markers also suggest possible translation‐to‐transcription feedback (i.e., the UPR and ER stress responses). Therefore, further research into increasing the threshold for this feedback could be a breakthrough for *Agrobacterium*‐mediated transient expression of recombinant proteins.

## 4 Materials and Methods

### 4.1 Experimental Structure

**Figure 6:**
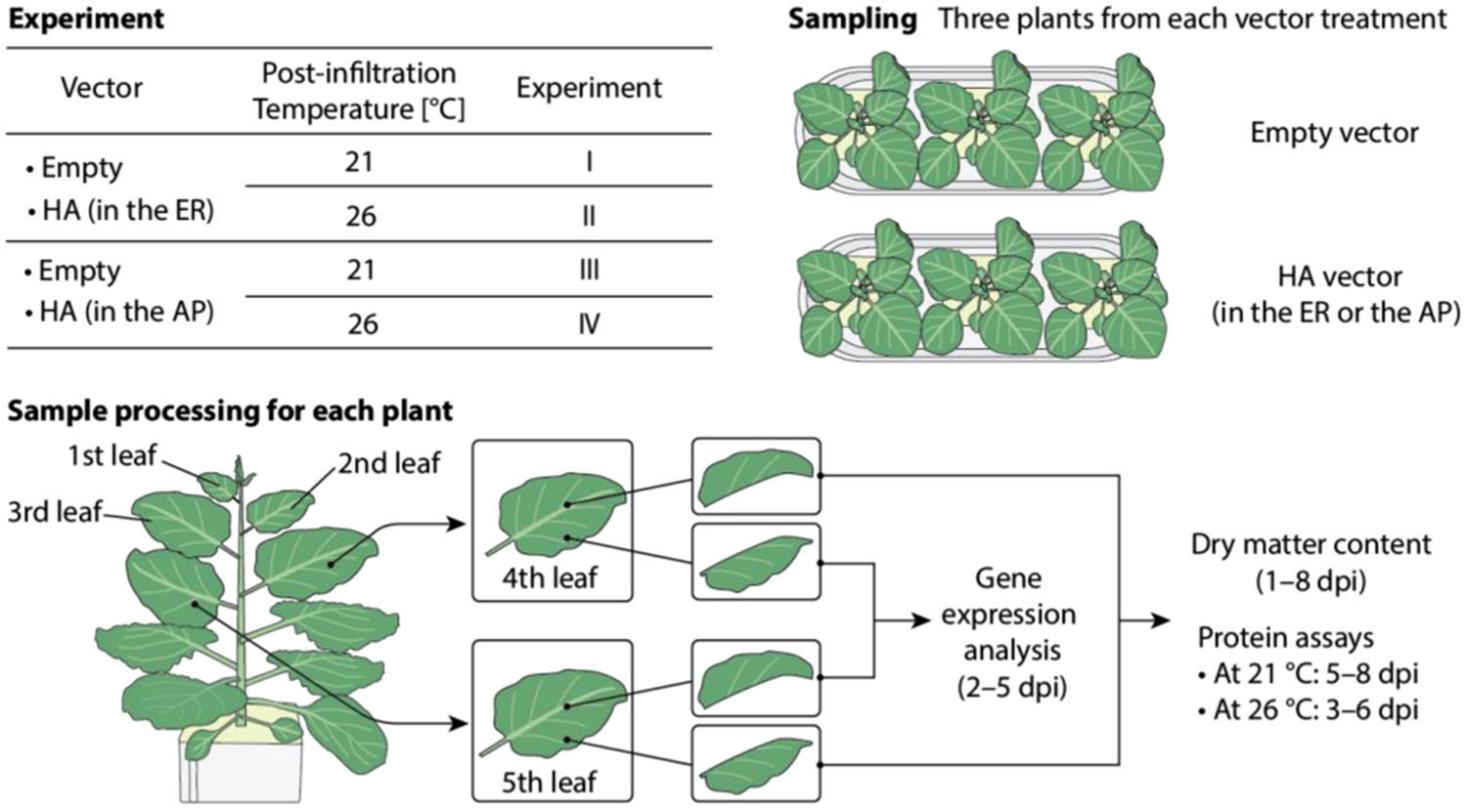
Experimental design and sampling scenarios.

This research consists of four experiments conducted separately (Figure 6). The *N. benthamiana* plants were introduced with one of the plasmid vectors, including those accumulating HA protein in the ER or the AP (Matsuda et al. 2017a) (Figure 6). Plants agroinfiltrated with an empty vector or HA vectors were grown at both 21°C and 26°C. The plants agroinfiltrated with an empty vector were used to normalize the gene expression levels of the respective temperature (Figure 6). We collected data from four experiments conducted separately as outlined in Table 1.

**Table 1:** Plasmid vectors and air temperature levels used in this research.

Experiment	Treatment code	Plasmid vector†	Preinfiltration Air temperature [°C]‡		Postinfiltration Air temperature [°C]§	
			Light period (16 h)	Dark period (8 h)	Light period (16 h)	Dark period (8 h)
1	215‐21	pNM215	25.7 ± 0.1	20.9 ± 0.1	20.7 ± 0.0	20.6 ± 0.0
	216‐21	pNM216				
2	215‐26	pNM215	25.9 ± 0.2	20.8 ± 0.3	26.0 ± 0.1	26.1 ± 0.0
	216‐26	pNM216				
3	215‐21	pNM215	26.1 ± 0.2	20.9 ± 0.6	20.0 ± 0.1	20.1 ± 0.1
	217‐21	pNM217				
4	215‐26	pNM215	26.0 ± 0.4	20.9 ± 0.5	25.7 ± 0.1	25.3 ± 2.1
	217‐26	pNM217				

^†^ pNM215: empty vector; pNM216: endoplasmic reticulum‐targeting hemagglutinin vector; pNM217: apoplast‐targeting hemagglutinin vector.

^‡^ Means were calculated using data collected from 0 to 37 dps.

^§^ Means were calculated using data collected from 38 to 46 dps.

For each experiment, we used two kinds of plants: plants with an HA vector (the ER‐targeting or the AP‐targeting) and those with an empty vector (the control we used for normalizing gene expression). We grew the plants at 21 or 26°C in each independent cultivation experiment. We collected three plants with an HA vector (*n* = 3) and three plants with an empty vector (*n* = 3) for each dpi. We harvested plants from 1 to 8 dpi for each experiment. In total, we harvested 48 plants from one experiment. Fundamentally, RNA transcription precedes protein translation. Thus, we measured transcript levels in the leaf samples harvested from 2 to 5 dpi, and we measured protein contents (HA and TSP) in the leaf samples harvested over the period from 3 to 6 dpi at 26°C or 5–8 dpi at 21°C.

### 4.2 Plant Material and Growth Conditions

The cultivation method was identical to that used in previous research (Matsuda et al. 2017a) except for the light source and air temperature settings. Briefly, cultivation before agroinfiltration (hereafter refer to as preinfiltration cultivation) of *N. benthamiana* plants from 0 day postseeding (dps)–37 dps was conducted in a temperature‐controlled growth chamber (MIR‐554‐PJ, Panasonic Co. Ltd. Osaka, Japan) equipped with phosphor‐converted white light‐emitting diode panels (GSPW1651NSE‐40Y‐TR, Stanley Electric Co. Ltd. Tokyo, Japan) (Supporting Information S1: Supplementary II). The light and dark periods were 16 and 8 h, respectively. During the light period, the average photosynthetic photon flux density (PPFD) was 200 ± 20 µmol m^–2^ s^–1^ at the top of the plants. The air temperature was controlled to an average of 26°C and 21°C over the light and dark periods, respectively. The air temperature was measured using calibrated T‐type thermocouples and recorded once a minute using a data logger (GL220, Graphtec Corporation, Yokohama, Japan) (Table 1). The subirrigation medium was tap water (0–6 dps) and subsequently changed to Prescription A nutrient solution (OAT No. 1 and No. 2; OAT Agrio Co. Ltd. Tokyo, Japan) with a pH of 6 and electrical conductivity of 0.18 S m^–1^.

Postinfiltration cultivation (38–46 dps) was continued in the growth chambers under the following environmental conditions. The same dark‐light cycle was maintained, but the PPFD was changed to 100 ± 5 µmol m^–2^ s^–1^ over the light period (Table 1). To test the effects of air temperature, the infiltrated plants were exposed to a constant air temperature of 21 or 26°C for 24 h d^–1^ over the postinfiltration period. The plants were subirrigated with tap water for 24 h d^–1^.

### 4.3 Expression Constructs and Vacuum Agroinfiltration

A tobamovirus‐derived “deconstructed” viral vector, magnICON (Icon Genetics GmbH, Halle/Saale, Germany) (Gleba, Klimyuk, and Marillonnet 2005; Marillonnet et al. 2005) was employed to express an ectodomain of HA (H1N1, A/California/07/2009 strain of influenza virus) (Figure 6). The N‐terminal of HA expressed in this research was modified to contain a secretory signal peptide derived from *Arabidopsis thaliana* auxin‐binding protein 1 (ABP1). We used two types of HA expression vectors. For HA targeted to the ER, its C‐terminal contains His‐Asp‐Glu‐Leu (HDEL) ER‐retention signal peptide. For HA targeted to the AP, its C‐terminal did not contain HDEL. The HA expression constructs, pNM216 and pNM217, contain *Arabidopsis thaliana* actin 2 promoter, RNA‐dependent RNA polymerase gene (*RdRP*), movement protein gene (*MP*), gene encoding ABP1‐HA‐HEDL (pNM216) or ABP1‐HA (pNM217), and Nos terminator. The expression construct without *HA* (pNM215) served as an empty vector (Figure 6). Details of the expression constructs were described in previous research (Matsuda et al. 2017a). Vacuum agroinfiltration was conducted at 38 dps using a previously described method (Matsuda et al. 2017a).

### 4.4 Leaf Sampling

Due to a time lag between transcription and translation, the sampling was conducted at 2–5 dpi for transcript level analysis. The sampling windows for HA and TSP contents were 5–8 dpi (21°C) and 3–6 dpi (26°C). These specific sampling windows were chosen based on previous research (Matsuda et al. 2017a) to obtain data on HA content before reaching the maximum, at the maximum, and after reaching the maximum.

At the time of sampling, each plant had approximately 11 true leaves. The fourth and fifth youngest fully expanded true leaves were collected from three plants per treatment combination at 1–8 dpi (Figure 6). Then, the leaves were cut into halves along the midvein (Figure 6). One half of the fourth and the fifth leaves, were pooled, immediately ground rapidly into a fine powder in liquid nitrogen, and used for transcript level analysis (Figure 6). The remaining two halves were cut into small pieces (ca. 6 mm × 6 mm), pooled, and divided into portions (Figure 6). Then, the fresh mass (FM) [g] of each portion was recorded immediately (Figure 6). One portion was used for DMC analysis (Figure 6). The rest were stored at –80°C for protein assays (Figure 6).

### 4.5 Dry Matter Content

The leaf samples collected at 1–8 dpi were used for DMC [g(DM) g(FM)^–1^] analysis following the previously described method (Matsuda et al. 2017a). Since leaves lost moisture along the development of necrosis, the DMC can be used as a quantitative indicator of the extent of necrosis (Matsuda et al. 2017a). On the sampling day, one portion of the leaf was immediately dried in a hot‐air oven at 100°C for 1 h and subsequently dried at 80°C for 3 days (Raguse and Smith 1965). Then, the dry mass (DM) [g] was recorded.

### 4.6 Protein Assays

The leaf samples collected at 5–8 dpi (21°C) and 3–6 dpi (26°C) were used for analyses of the TSP and HA contents. The sampling window for 21°C and 26°C were determined differently for the protein data covering three stages, before the maximum, at the maximum, and after the maximum (Matsuda et al. 2017a).

The HA content was quantified by following the protocol previously described by Fujiuchi et al. (2016). Briefly, we used a sandwich ELISA kit (SEK001, Sino Biological Inc. China). A standard protein was recombinant influenza A H1N1 HA. A capture antibody was mouse anti‐influenza A H1N1 HA monoclonal antibody. A detection antibody was rabbit anti‐influenza A H1N1 HA polyclonal antibody conjugated to horseradish peroxidase (HRP). We detected the activity of HRP with its substrate solution by measuring the absorbance at 450 nm of its product and created a standard curve. The absorbance at 450 nm of the samples was subsequently converted to HA content using the standard curve of the standard HA protein and reported as mg g(DM)^–1^.

The TSP content was quantified using the Bradford colorimetric assay using an assay kit (Bio‐Rad Laboratories Inc. California, USA), as previously described (Fujiuchi et al. 2016). The supernatant of leaf homogenate was diluted fivefolds in distilled water. Dilutions of standard bovine serum albumin (Bio‐Rad Laboratories Inc.) were used to create a standard curve. Samples and standard dilutions were added with Bradford reagent (Protein Assay Dye Reagent, Bio‐Rad Laboratories Inc.). and incubated for 5 min before measuring absorbance at 595 nm. With reference to the standard curve, TSP content was calculated and reported as mg g(DM)^–1^.

### 4.7 Transcript Level Analysis

The fine powder of leaf samples collected at 2–5 dpi was used for transcript level analysis. Total RNA was isolated from leaf powder using a total RNA isolation kit (RNAqueous Phenol‐free total RNA isolation kit, Thermo Fisher Scientific Inc. Massachusetts, USA). Contaminant DNA was removed using a kit (TURBO DNA‐free Kit, Thermo Fisher Scientific Inc.) according to the manufacturer's instructions. Total RNA samples with an OD_260/280_ ratio within the range of 2.0–2.1 and an OD_260/230_ ratio within the range of 2.1–2.3 were used for preparing cDNA with a kit (High‐capacity cDNA Reverse Transcription Kit, Thermo Fisher Scientific, USA). The cDNA samples were stored at –20°C until analysis (Hamorsky et al. 2015).

Transcript levels of the genes of interest were quantified using real‐time PCR as described previously (Hamorsky et al. 2015) under the conditions shown in Supporting Information S1: Supplementary I. The transcript level in leaves localizing HA in the ER or AP at 21°C and 26°C from 2 to 5 dpi compared to those in the empty vector‐infiltrated plants was calculated using the mean *C*_q_ of the two technical replicates. The housekeeping gene *18S* was used as a reference gene. The transcript levels were normalized using data obtained from the plants infiltrated with the empty vector at the same temperature, except those of the *HA* gene.

### 4.8 Statistical Analysis

R (version 1.4.1106) was used for statistical processing for all analyses. To clarify how temperature affected the changes in HA content and TSP content over time (i.e., the number of days needed to reach the peak and the reduction rate) and the changes in DMC content over time (i.e., the progression of necrosis), the significant differences in means among dpi in each temperature treatment were tested by one‐way analysis of variance (ANOVA) followed by Tukey's honestly significant difference test (*p* < 0.05) in the R package “agricolae.” In the gene expression level analysis, to clarify the impact of 21°C and 26°C on ER stress and HR levels, the significant differences in the means of gene expression levels at the same dpi between the two temperature levels were tested by Welch's *t*‐test at *p* < 0.05. Three plant samples were harvested for each treatment per dpi, and individual plants were used as experimental units (*n* = 3).

## Author Contributions

All authors conceptualized the aim of this article. P.P., K.A., and R.M. designed experiments. P.P. conducted the experiments. P.P. wrote the manuscript. R.M., N.M., K.A., and Y.O. critically reviewed and revised the manuscript. R.M. acquired funding. All authors approved the final manuscript and agreed to be accountable for all aspects of the manuscript.

## Supporting information

Supporting information.

## Data Availability

The data that support the findings of this study are available from the corresponding author upon reasonable request.
