# Supplementary material for: Effects of Air Temperature on Transient Expression of Influenza Hemagglutinin in Nicotiana benthamiana: Analysis of Transgene Transcription and Plant Stress Responses
Source: Biotechnol Bioeng. 2025 Jan 31;122(5):1142–52. doi: 10.1002/bit.28942 (PMC11975175; doi:10.1002/bit.28942)
Supplement: Supplementary file 1 — Supporting information. [file BIT-122-1142-s001.docx]

**Supplementary I.** List of genes used in RT-qPCR with annealing temperature and validation of qPCR shown by the slope, *y*-intercept, efficiency (*E*), and *r*^2^ of the calibration curves.

| **Gene** | **Gene Name** | **RT-qPCR Sequences**  **Forward (F) and Reverse (R)** | **Annealing Temperature (°C)** | **Slope** | ***y*-intercept** | ***r*^2^** | ***E* (%)** |
| --- | --- | --- | --- | --- | --- | --- | --- |
| *MP* | Movement protein | **F** 5’-TCG ATT CGG TTG CAG CAT TTA-3’  **R** 5’-CGT ACT TCT CCG GTC TGT ACT T-3’ | 65 | -3.484 | 19.42 | 0.996 | 93.4 |
| *RdRP* | RNA-dependent RNA polymerase | **F** 5’-ACT CTG TTC CGT GGT GTG TAC-3’  **R** 5’-ACG CAG CGT TAT CCT TGA AG-3’ | 55 | -3.715 | 20.62 | 0.997 | 85.7 |
| *HA* | Hemagglutinin | **F** 5’-GTG GAT ACC GTG CTT GAG AAG-3’  **R** 5’-ACC AAG AAT CCA ACC AGC G-3’ | 55 | -3.280 | 23.51 | 0.993 | 101.9 |

**Supplementary II.** Relative spectral photosynthetic photon flux density distribution of white LED light used as growth light measured by using a spectroradiometer (MS-720; EKO Instruments Co. Ltd., Tokyo, Japan)
